# How many cases of disease in a pedigree imply familial disease?

**DOI:** 10.1111/ahg.12222

**Published:** 2017-10-23

**Authors:** Frank Dudbridge, Suzanne J. Brown, Lynley Ward, Scott G. Wilson, John P. Walsh

**Affiliations:** ^1^ Department of Health Sciences University of Leicester Leicester UK; ^2^ Department of Endocrinology & Diabetes Sir Charles Gairdner Hospital Nedlands Australia; ^3^ School of Medicine and Pharmacology University of Western Australia Crawley Australia; ^4^ Department of Twin Research & Genetic Epidemiology King's College London London UK

**Keywords:** familial disease, family history, rare variants, thyroid cancer, whole genome sequencing

## Abstract

The ability to perform whole‐exome and, increasingly, whole‐genome sequencing on large numbers of individuals has led to increased efforts to identify rare genetic variants that affect the risk of both common and rare diseases. In such applications, it is important to identify families that are segregating the rare variants of interest. For rare diseases or rare familial forms of common diseases, pedigrees with multiple affected members are clearly harbouring risk variants. For more common diseases, however, it may be unclear whether a family with a few affected members is segregating a familial disease, is the result of multiple sporadic cases, or is a mixture of familial cases and phenocopies. We provide calculations for the probability that a family is harbouring familial disease, presented in general terms that admit working guidelines for selecting families for current sequencing studies. Using examples motivated by our own studies of thyroid cancer and published studies of colorectal cancer, we show that for common diseases, families with exactly two affected first‐degree relatives have only a moderate probability of segregating familial disease, but this probability is higher for families with three or more affected relatives, and those families should therefore be prioritised in sequencing studies.

## 1 INTRODUCTION

In recent years, the ability to perform whole‐exome and, increasingly, whole‐genome sequencing on large numbers of individuals has led to increased efforts to identify rare genetic variants that affect the risk of disease (Goodwin, Mcpherson, & Mccombie, 2016). In the case of rare Mendelian diseases, sequencing has obviated the need for linkage and fine mapping analyses in pedigrees, allowing more direct identification of causal variants. For common diseases, familial forms may exist that can be regarded as Mendelian disorders, or there may be rare variants with reduced penetrance that account for some familial aggregation and explain some of the missing heritability not identified by genome‐wide association studies of common variation.

In each of these applications, it is important to identify families that are segregating the rare variants of interest. For rare diseases or familial forms, pedigrees with multiple affected members are clearly harbouring risk variants. For example, a study of Waldenström macroglobulinemia identified two novel candidate genes by exome sequencing three affected and one unaffected members of one pedigree, together with 50 unrelated cases with a family history and 196 cases without (Roccaro et al., 2016). Similarly, a study of familial bipolar disorder identified a number of candidate genes by exome sequencing eight families, originally ascertained for linkage studies, each with at least four affected members (Goes et al., 2016). Similar strategies have been successful for other rare phenotypes (Preuss et al., 2016; Braun et al., 2016).

For more common diseases, however, it may be unclear whether a family with a few affected members is segregating a single variant with incomplete penetrance, is the result of multiple sporadic (or polygenic) cases, or includes a mixture of familial cases and phenocopies. For example, a recent study of colorectal cancer performed exome sequencing in 1006 early‐onset cases with at least one affected first‐degree relative but could only ascribe 16% of familial disease to highly penetrant rare mutations (Chubb et al., 2016). On the other hand, in another study of colorectal cancer in which cases were selected to have at least three affected relatives, 22 of the 29 families showed evidence for segregating variants (Esteban‐Jurado et al., 2015).

Our own study aimed to find rare variants causing familial nonmedullary thyroid cancer, a rare form of differentiated thyroid cancer, using a series of families, the majority having two or three affected individuals (Weeks et al., 2016). With an estimated lifetime risk less than 0.02 (Threlfall & Thompson, 2015), thyroid cancer is not common but is not as rare as many Mendelian diseases (which typically have a prevalence below 1 in 2000). However, familial disease is estimated to be present in only about 5% of thyroid cancer cases (Khan, Smellie, Nutting, Harrington, & Newbold, 2010). Previously it has been of interest to identify familial cases in order to study clinical characteristics of the disease such as its increased local invasiveness. Calculations by Charkes (2006) suggested that only 60.6% of families with two or more affected individuals are harbouring the familial form of this disease, but when there are three or more affected members, almost all families have the familial form. Such calculations are also relevant for informing the efficient selection of families for sequencing studies. Of course, formal segregation analysis may be applied to any given family to determine the likelihood of it segregating a rare variant, but such calculations can be challenging in a clinical environment, in which it may be more useful to have practical guidelines for identifying families worth following up more closely. Our aim here is to provide calculations for the probability that a family is harbouring a familial form of disease and to present these calculations in general terms that admit working guidelines for selecting families for the current generation of sequencing studies. Our approach is similar to that of Charkes (2006), but we consider ascertainment more explicitly and correct an error in a probability calculation, leading to quantitatively different results from those previously reported.

## 2 METHODS

Let us distinguish between families that harbour a presumed disease mutation (“F‐families”) or do not (“R‐families”). Let $p_{R}$ be the risk of sporadic disease; if the familial form is rare, then $p_{R}$ is approximately the population risk. For the present purposes, we include polygenic effects in the sporadic risk. Also let $p_{F}$ be the risk of familial disease for individuals in an F‐family, excluding the proband. This quantity depends on both the penetrance of the presumed mutation and the relationship of individuals to the proband. Here, we will only consider the first‐degree relatives of the proband, so, for example, $p_{F}$ can be taken as 0.5 for a fully penetrant dominant mutation. For more general pedigrees, $p_{F}$ could be understood as an average over the individuals considered; though less precise than a proper segregation analysis, we shall see that this simple parameterisation allows ready calculation of practical guidelines for identifying F‐families.

We want the probability that a family of size *k* with *m* cases, excluding the proband, is an F‐family. Assuming that family size is independent of whether a family is an F‐ or R‐family,

(1) $$\mathrm{Pr}\left(F\;|\;m;k\right)=\frac{\mathrm{Pr}(m\;|\;F;k)\mathrm{Pr}(F)}{\mathrm{Pr}(m\;|\;F;k)\mathrm{Pr}(F)+\mathrm{Pr}(m\;|\;R;k)\mathrm{Pr}(R)}$$

For F‐families, the probability of observing *m* cases is the probability of observing $m_{F}$ familial and $m-m_{F}$ sporadic cases, summed over $m_{F}=0,\ldots ,m$. The probability of $m_{F}$ familial cases in an F‐family of size *k* is given by the binomial distribution as

$$\left(\begin{array}{c} k\\ m_{F} \end{array}\right)p_{F}^{m_{F}}{\left(1-p_{F}\right)}^{k-m_{F}},$$

and the probability of $m-m_{F}$ sporadic cases among the remaining $k-m_{F}$ family members is

$$\left(\begin{array}{c} k-m_{F}\\ m-m_{F} \end{array}\right)p_{R}^{m-m_{F}}{\left(1-p_{R}\right)}^{k-m}.$$

Therefore,

(2) $$\begin{array}{ccc} \mathrm{Pr}(m\;|\;F;k) & = & \sum_{m_{F}=0}^{m}\left(\begin{array}{c} k\\ m_{F} \end{array}\right)p_{F}^{m_{F}}{\left(1-p_{F}\right)}^{k-m_{F}}\\ & & \times \left(\begin{array}{c} k-m_{F}\\ m-m_{F} \end{array}\right)p_{R}^{m-m_{F}}{\left(1-p_{R}\right)}^{k-m} \end{array}$$

(This formula corrects an error in the power of $\left(1-p_{R}\right)$ in the Appendix of Charkes [2006]).

For R‐families, only sporadic cases can be observed, and so

(3) $$\mathrm{Pr}\left(m\;|\;R;k\right)=\left(\begin{array}{c} k\\ m \end{array}\right)p_{R}^{m}{\left(1-p_{R}\right)}^{k-m}$$

To calculate Pr(F|m;k) then, we require values for $p_{F}$, $p_{R}$, and the proportion of F‐families Pr(F). These can often be estimated from epidemiological data, as we show below.

## 3 RESULTS

We revisit the example of thyroid cancer presented by Charkes (2006). The lifetime risk was estimated from cancer registry data as $p_{R}=0.0031$. The risk of familial disease in F‐families was calculated from families with at least three affected first‐degree relatives, for which it will be shown that the vast majority contain three familial cases. Charkes (2006, Table 2) reported 23 cases among 72 subjects in 7 families, from which a naïve estimate of $p_{F}$ was taken as 23/72 = 0.319. As noted however, this estimate does not account for ascertainment. To obtain a more accurate estimate, accounting for selection of families with at least two affected first‐degree relatives of the proband, we define a likelihood for $p_{F}$ as the binomial probability of *m* cases in a family of size *k*, excluding the proband, divided by the cumulative probability of at least two such cases. Over the seven families tabulated by Charkes, numerical maximisation of this likelihood gives our ascertainment‐corrected estimate as $p_{F}=0.0981$.

To estimate the proportion of F‐families Pr(F), we use the fact that of 8214 affected families tabulated by Charkes (2006, Table 1A), 260 had at least two cases. In R‐families, the probability of at least one additional case to the proband is

$$\sum_{m=1}^{k}\left(\begin{array}{c} k\\ m \end{array}\right)p_{R}^{m}{\left(1-p_{R}\right)}^{k-m}$$

Assuming an average number of k=8 first‐degree relatives of the proband and using $p_{R}=0.0031$ as estimated from cancer registry data, this probability is 0.0245. In F‐families, Equation (2) may be summed in the same way, using $p_{F}=0.0981$ as estimated above, to obtain the corresponding probability as 0.573. The total proportion of families with one additional case is then

$$\frac{260}{8214}=0.573\mathrm{Pr}\left(F\right)+0.0245\mathrm{Pr}\left(R\right)$$

with the solution Pr(F)=0.013, Pr(R)=0.987. For a family with eight first‐degree relatives of the proband, of which just one is affected, we use Equations (1) and (2) to obtain

Pr(F|m=1;k=8)=0.172

Thus, under these parameters, there is only a roughly 1 in 6 chance that such a family is segregating a familial form of disease. This is somewhat lower than the figure of 0.323 previously calculated for the same data (Charkes, 2006), although both results suggest that an F‐family is unlikely.

**Figure 1:**
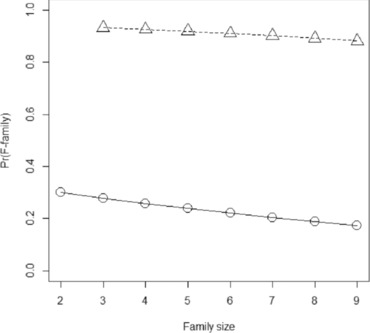
Probability of an F‐family for differentiated thyroid cancer. Family size, total size of family including proband and first‐degree relatives with known affection status (= *k *+ 1). *Circles* and *solid line*, proband has exactly *m* = 1 affected first relative. *Triangles* and *dashed line*, proband has two affected first degree relatives

Figure 1 shows the probability that a family with exactly one additional case is an F‐family, for different family sizes. For larger families, the probability of familial disease is lower, because if it were present we should expect a higher number of cases. Conversely, for smaller families, the higher proportion of cases is more consistent with familial disease. Therefore, the probability of familial disease depends critically on the number of family members whose disease status can be ascertained.

For families with two additional cases, the probability of an F‐family is 0.882 for k=8 , and over 90% for smaller families (Figure 1). For families with three additional cases, there is over 99% probability of an F‐family for all family sizes up to k=8. Therefore, as claimed above, families with at least three cases may be safely assumed to be F‐families. Among such families, we can calculate the chance that all affected relatives have the familial form of disease. This is

$$\begin{array}{c} \frac{\left(\begin{array}{c} k\\ m \end{array}\right)p_{F}^{m}{\left(1-p_{F}\right)}^{k-m}{\left(1-p_{R}\right)}^{k-m}}{\sum_{m_{F}=0}^{m}\left(\begin{array}{c} k\\ m_{F} \end{array}\right)p_{F}^{m_{F}}{\left(1-p_{F}\right)}^{k-m_{F}}\left(\begin{array}{c} k-m_{F}\\ m-m_{F} \end{array}\right)p_{R}^{m-m_{F}}{\left(1-p_{R}\right)}^{k-m}}, \end{array}$$

in which the numerator is the probability that all *m* additional cases have familial disease, with the remaining *k‐m* relatives unaffected, and the denominator is the probability of *m* cases in an F‐family as given by Equation (2).

The probability that all affected relatives have familial disease is 0.945 when *m* = 2 and remains high at 0.799 even when all eight relatives are affected. In the example of thyroid cancer, then, a general guideline might be that families with at least two affected first‐degree relatives of the proband are highly likely to be segregating familial nonmedullary thyroid cancer. In such families, it is highly likely that all cases have the familial form.

For comparison, we repeat these calculations for colorectal cancer, a more common condition. Recall that Chubb et al. (2016) sequenced early‐onset cases with at least one affected first‐degree relative. We will calculate the probability that these pedigrees have familial disease. A rough estimate of $p_{F}$ can be obtained from another recent study of pedigrees with three or more affected relatives, two or more consecutive affected generations, at least one early‐onset case, and segregation consistent with autosomal dominance (Esteban‐Jurado et al., 2015). In each of the 29 pedigrees described, we selected an index case and its first‐degree relatives such that the number of affected relatives was maximised. This led to a total of 68 affected relatives out of 241, and an ascertainment‐naïve estimate of $p_{F}=0.282$. Since the ascertainment was more strict than in Chubb et al., we attenuated this to $p_{F}=0.2$ to allow for selection of less clearly familial pedigrees. Finally, assume the lifetime risk of $p_{R}=0.05$ (Esteban‐Jurado et al., 2015).

Chubb et al. (2016) identified penetrant rare mutations in 16% of families with at least one affected first‐degree relative. Allowing for incomplete power in that study, suppose

Pr(F|m≥1)=0.2. From Bayes’ theorem,

$$\mathrm{Pr}\left(F\;|\;m\geq 1\right)=\frac{\sum_{m=1}^{k}\mathrm{Pr}(m\;|\;F)\mathrm{Pr}\left(F\right)}{\sum_{m=1}^{k}\mathrm{Pr}(m\;|\;F)\mathrm{Pr}\left(F\right)+\mathrm{Pr}\left(m\;|\;R\right)\mathrm{Pr}\left(R\right)}=0.2$$

Using Equations (2) and (3) to evaluate the sums assuming k=8, we solve to obtain Pr(F)=0.0865. Equation (1) then gives Pr(F|m=1;k=8)=0.0870.

**Figure 2:**
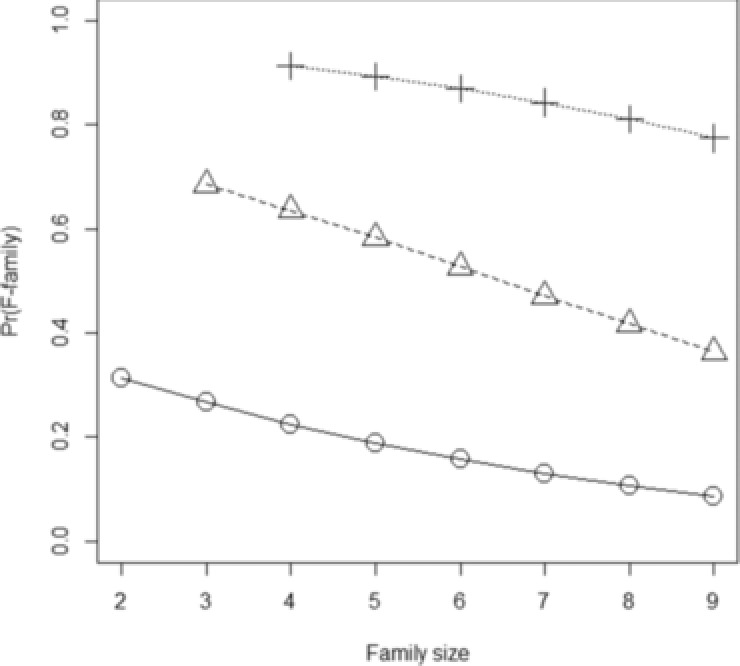
Probability of an F‐family for colorectal cancer. Family size, total size of family including proband and first‐degree relatives with known affection status (= *k *+ 1). *Circles* and *solid line*, proband has exactly *m* = 1 affected first relative. *Triangles* and *dashed line*, proband has two affected first‐degree relatives. *Crosses* and *dotted line*, proband has three affected first‐degree relatives

Thus, the chance of such a family having familial disease is lower than for thyroid cancer, owing to the higher sporadic risk of disease. Figure 2 shows the probability of an F‐family for different family sizes and numbers of affected relatives. For two affected relatives, the probability of an F‐family is still only 36.4%, but increases to over 77% for three or more relatives.

For k=8 relatives, the probabilities that all cases in an F‐family are familial are lower than for our example of differentiated thyroid cancer, ranging from 0.694 for m=2 to 0.233 for m=8. However, allowing for one sporadic relative per family gives higher probabilities of the remainder being familial: 0.972 for m=2 to 0.605 for m=8. Overall then, similar conclusions apply for colorectal cancer as for thyroid cancer: probands with only one affected relative have only moderate probability of being an F‐family, but those with two or more affected relatives have high probability of being an F‐family, and nearly all of those cases are familial. Thus, for both of these diseases, a good rule for identifying rare familial mutations would be to sequence families with at least two affected first‐degree relatives of the proband.

## 4 DISCUSSION

Our approach differs from that of Charkes (2006) in several ways. Rather than considering all possible configurations of cases in a family, we have omitted the proband from the calculations and considered only its relatives. We believe the latter to be more appropriate because within any pedigree the set of relevant (e.g., first‐degree) relatives depends on the identity of the proband, and thus the binomial trial design itself is conditional on the proband. Formula (2) corrects an error in the power of $\left(1-p_{R}\right)$ in the Appendix of Charkes (2006). Finally, we have more explicitly allowed for ascertainment in estimating $p_{F}$. However, the conclusions remain very similar under our improved calculations. Note that we assumed that family size is independent of familial disease, which may be untrue in reality. Furthermore, the distinction between familial and sporadic disease is less clear when searching for low‐penetrance variants. To estimate the proportion of F‐families, epidemiological data on multiply affected pedigrees can be used, but they may not be representative of the smaller pedigrees arising from reduced penetrance. Nevertheless, our results suggest that for both rare and common disease, probands should have at least two affected first‐degree relatives for there to be a high chance that the family is segregating a penetrant rare variant. A spreadsheet implementing our calculations is provided as Supporting Information and a more detailed R package is available from https://github.com/DudbridgeLab/probFamilial/.

## Supporting information

Table S1
